# Increase in Cerebellar Volume in Cavalier King Charles Spaniels with Chiari-like Malformation and Its Role in the Development of Syringomyelia

**DOI:** 10.1371/journal.pone.0033660

**Published:** 2012-04-10

**Authors:** Thomas A. Shaw, Imelda M. McGonnell, Colin J. Driver, Clare Rusbridge, Holger A. Volk

**Affiliations:** 1 Department of Veterinary Clinical Sciences, Royal Veterinary College, Hatfield, Hertfordshire, United Kingdom; 2 Department of Veterinary Basic Sciences, The Royal Veterinary College, London, United Kingdom; 3 Stone Lion Veterinary Hospital, Wimbledon, London, United Kingdom; Tokyo Medical and Dental University, Japan

## Abstract

Previous research in Cavalier King Charles Spaniels (CKCS) has found that Chiari-like malformation and syringomyelia (CM/SM) are associated with a volume mismatch between the caudal cranial fossa (CCF) and the brain parenchyma contained within. The objectives of this study were to i) compare cerebellar volume in CKCS (a “high risk’ group which frequently develops CM/SM), small breed dogs (medium risk – occasionally develop CM/SM), and Labradors (low risk – CM/SM not reported); ii) evaluate a possible association between increased cerebellar volume and CM/SM in CKCS; iii) investigate the relationship between increased cerebellar volume and crowding of the cerebellum in the caudal part of the CCF (i.e. the region of the foramen magnum). Volumes of three-dimensional, magnetic resonance imaging derived models of the CCF and cerebellum were obtained from 75 CKCS, 44 small breed dogs, and 31 Labradors. As SM is thought to be a late onset disease process, two subgroups were formed for comparison: 18 CKCS younger than 2 years with SM (CM/SM group) and 13 CKCS older than 5 years without SM (CM group). Relative cerebellar volume was defined as the volume of the cerebellum divided by the total volume of brain parenchyma. Our results show that the CKCS has a relatively larger cerebellum than small breed dogs and Labradors and provide evidence that increased cerebellar volume in CKCS is associated with crowding of cerebellum in the caudal part of the CCF. In CKCS there is an association between increased cerebellar volume and SM. These findings have implications for the understanding of the pathological mechanisms of CM/SM, and support the hypothesis that it is a multifactorial disease process governed by increased cerebellar volume and failure of the CCF to reach a commensurate size.

## Introduction

Chiari-like Malformation (CM) and syringomyelia (SM) is a debilitating and painful disease complex in the Cavalier King Charles Spaniel dog breed (CKCS) which is regarded as a complex oligogenic trait of moderately high heritability [1], [2], [3], [4]. CM is characterised by foramen magnum cerebellar herniation [5], [6] and occurs in approximately 95% of the CKCS population [7]. More than half of CKCS over 4 years of age have SM, fluid filled cavities within the spinal cord (syringes) [8]. A causal relationship between CM and SM has been hypothesised, the pathophysiology of which is presumed to be mediated by abnormal cerebrospinal fluid flow dynamics [9], [10]. CM/SM in the CKCS is commonly associated with pain, especially in the cervical region, and with various neurological dysfunctions such as scoliosis, limb paresis and ataxia [5], [11]. Affected dogs might be hypersensitive to touch and often scratch an area on the shoulder, ear, neck or sternum, commonly only on one side of the body and without making skin contact (‘phantom scratching’) [11]. Some dogs perform facial or head rubbing or spontaneous vocalisations.

In humans, ‘Classical’ Chiari type I malformation is believed to result from hypoplasia of the basioccipital bone and a consequent reduction in the volume of the posterior cranial fossa (which extends from the petrosal crests and dorsum sellae to the foramen magnum), leading to overcrowding by hindbrain parenchyma (cerebellum, pons and medulla oblongata) and herniation of the cerebellum through the foramen magnum [12], [13], [14]. Several morphometric analyses have been conducted on the CKCS caudal cranial fossa (CCF), which is homologous to the posterior cranial fossa in humans [15]. As with Classical Chiari type I malformation in humans, CKCS appear to have a shallower caudal cranial fossa and have abnormalities of the supraoccipital and basioccipital bones when compared to mesaticephalic breeds (breeds with a skull of intermediate length and width) [16]. However, although CCF overcrowding has been demonstrated in CKCS with CM/SM, independent studies have reported no difference in relative CCF volume in CKCS compared to brachycephalic breeds or other small breed dogs [17], [18], or a link between CCF volume and the development of SM [19], [20], [21]. In contrast, a recent study has showed that there is evidence of increased volume of brain parenchyma within the CCF in CKCS with syringomyelia compared to CKCS without syringomyelia [19]. A separate study of volumetric breed comparisons showed that the relative volume of hindbrain parenchyma in CKCS is greater than in other small breed dogs (it is approximately equal to the Labrador - a mesaticephalic breed) [18]. These are interesting findings as the volume of parenchyma in the CCF maintains a consistent ratio to the volume of other brain regions in normal dogs [22].

### Aims of study

These previous studies did not identify which of the sub-divisions of the hindbrain, if any, were enlarged in CKCS with syringomyelia. Our hypotheses are:

- cerebellar volume is larger in CKCS than in other breed groups.
- increased cerebellar volume in CKCS is associated with syringomyelia.

We conducted a retrospective volumetric study of magnetic resonance (MR) images from small breed dogs, Labradors and CKCS with CM to compare the volume of the cerebellum and the remaining hindbrain (i.e. brainstem) in these groups. We chose to compare these breed groups as they represent “high risk” (CKCS have a high rate of CM/SM), “medium risk” (small breed dogs may occasionally develop CM/SM [2], [3], [23]) and “low risk” (CM/SM has not been recorded in the Labrador). Additionally, CKCS with CM and SM were compared to CKCS with CM but not SM. Our findings support the hypotheses that CKCS have increased cerebellar volume compared to other breeds and that increased cerebellar volume in CKCS is linked to the development of SM.

Chiari-like malformation in CKCS may be due to a mis-match between the volume of the cerebellum and the CCF, leading to cerebellar crowding in the caudal CCF and consequent herniation of cerebellar tonsils through the Foramen Magnum. We further hypothesise that:

- Increased cerebellar volume in CKCS is correlated with increased crowding of the cerebellum in the caudal part of the CCF.

We studied this by dividing the CCF into rostral and caudal sections to investigate the crowding of the cerebellum within each part. We found that increased cerebellar volume in the CKCS is correlated with crowding of the cerebellum in the caudal CCF and this is not seen in Labradors or small breed dogs. These results have implications for the understanding of the pathological mechanisms of CM/SM in CKCS.

## Materials and Methods

### Subjects

Dogs were retrospectively selected from Electronic patient records (Rx Works 4.2.1560, RxWorks Inc., 2010) from May 2004 to August 2010 (stored at the Royal Veterinary College and Stone Lion Veterinary Hospital) and assessed for suitability by reviewing T2-weighted transverse and mid-sagittal MR imaging scans using Image Viewer software (Image Viewer 4.0.18, Visbion, 2009) according to the following criteria:

- Transverse scans had to have included the cribiform plate rostrally and the first cervical spinal cord segment caudally.
- Breeds were selected according to the same criteria as a previous volumetric study on these groups [18]: CKCS, Labrador or a small breed from the toy or utility groups as defined by the Kennel Club; all skeletally mature (over 8 months of age in CKCS and SB, over 12 months of age in Labradors).
- Dogs with parenchymal space-occupying lesions or other conditions thought likely to raise intracranial pressure were excluded.
- Midsagittal scans had to have included the first three vertebrae of the cervical spine.
- CM was defined as evidence of caudal cerebellar herniation into the foramen magnum or indentation by the supraoccipital bone, irrespective of the presence of SM [6].
- A syrinx was defined as a fluid-containing cavity within the spinal cord parenchyma with a transverse diameter of greater than or equal to 2 mm [24].

Subjects were divided into the following groups: Small breed dogs (SB), Labradors (LD) and CKCS. CCF, cranial cranial fossa, and brain parenchyma volumes of all SB and LD individuals and 42 CKCS were used in a previous study [18]. CCF, cranial cranial fossa and brain parenchyma volumes of 49 CKCS individuals were used in another study [19]. SM is thought to be a late onset disease, and previous studies which did not select CKCS according to age may have failed to distinguish dogs that develop SM later in life from ‘true’ normal dogs that are unlikely to ever develop the condition. CKCS were therefore further subdivided into two groups based on age and presence or absence of SM: the CM/SM group which comprised eighteen individuals under the age of 2 years with SM, all of which presented with clinical signs related to syringomyelia, and thirteen individuals over the age of 5 years with CM but without SM (the CM group), which presented for various reasons including idiopathic epilepsy (n = 4), intervertebral disc disease (n = 3), otitis media with effusion (n = 2), MR imaging screening programme for breeding (n = 3) and facial nerve paralysis (n = 2). While the relationship between age and brain atrophy in humans is well documented and could potentially bias a comparison between different age groups, there is no evidence that this is the case in dogs (a recent study has shown that Labradors in the age groups 1–5, 5–10 and 10+ have similar cerebellar volumes) [22] or even chimpanzees [25]. We nevertheless conducted an analysis of cerebellar volume and age in SM-negative CKCS to see if this was a potential source of bias.

Approval from the ethics committee of the Royal Veterinary College was not sought as it is the policy of the ethics committee not to subject retrospective studies of images stored in the archive to ethical review.

### MR image analysis

**Figure 1:**
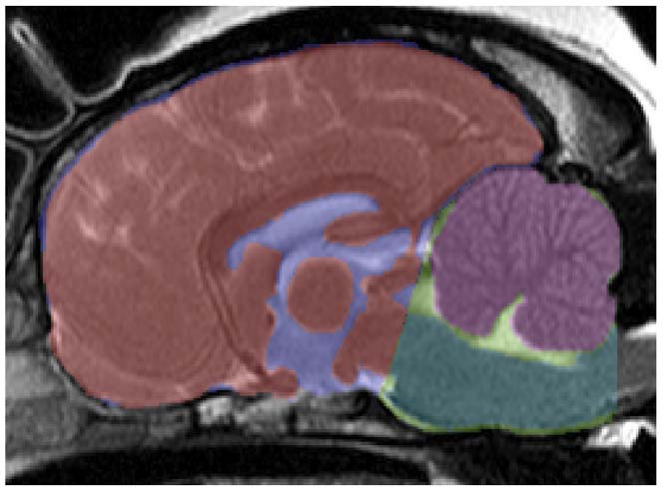
Masks recorded from MR images. Masks were recorded for the following volumes (mid-saggital view): Parenchyma within the cranial cranial fossa (red), cerebellum (purple), brainstem (dark green), CCF (light green). A mask for the cranial cranial fossa was not recorded but is shown here for completeness (blue).

The MR imaging report of a board-certified radiologist was consulted for each patient. Suitable series of transverse T2-weighted MR images (Philips NT Intera 1.5T MRI, 3 mm slice thickness, 0 mm interslice gap) were exported to a 3-D modelling software program (Mimics® 13.10, Materialise n.v., 2009) and measured as described in detail in previous volumetric studies [18], [19]. Study numbers were assigned to dogs so that observers were blinded to breed, age and the presence of SM. Image threshold was set to zero and contrast altered to include all brain parenchyma. Masks were created in two dimensions from individual slices using a pen tablet (XP-Pen® v. 4.04, P-Active Co. Ltd., 2003) for accuracy of free-hand measurements and the following volumes recorded (see Figure 1):

- Parenchyma within the cranial cranial fossa (a)
- Parenchyma within the CCF was measured as:
  • Cerebellum (b)
  • Brainstem (c)
  ○ CCF (d)

**Figure 2:**
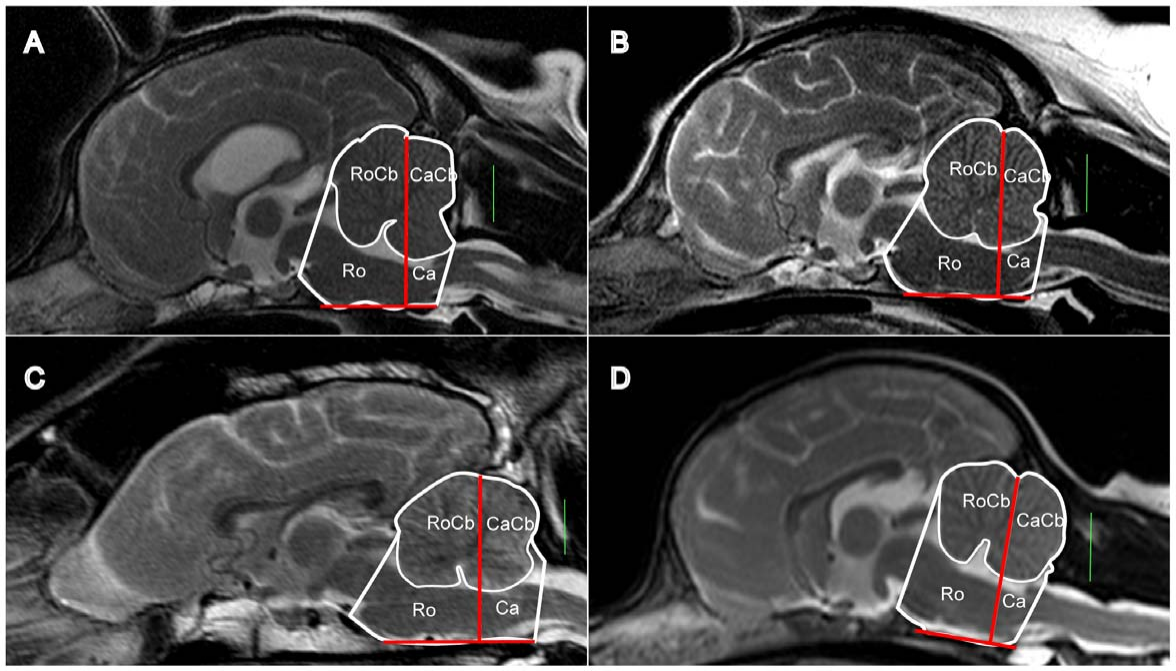
Partitioning of the CCF and cerebellum (mid-saggital view). A: CKCS - CM/SM group (top left), B: CKCS - CM group (top right), C: Labrador (bottom left), D: small breed dog – Chihuahua (bottom right). Masks were recorded for the following volumes: rostral CCF (Ro), caudal CCF (Ca), rostral cerebellum (RoCb – superimposed on Ro) and caudal cerebellum (CaCb – superimposed on Ca). The green bar is a 1 cm scale. The Red bars represent the division of the CCF into a caudal part and a rostral part by a plane orthogonal to the sagittal images, intersecting the base of the internal occipital protuberance and orientated perpendicular to the basioccipital bone.

Three-dimensional STL models of the CCF and cerebellum obtained from the masks described above were incorporated into Mimics projects containing sagittal scans of the subjects and aligned with the images. Subjects with missing or incomplete sagittal series were discarded from this part of the study. The CCF was divided into a caudal part and a rostral part by a plane orthogonal to the sagittal images, intersecting the base of the internal occipital protuberance and orientated perpendicular to the basioccipital bone (see Figure 2). Volumes were recorded for the following:

- cerebellum within the caudal part of the CCF (e)
- cerebellum within the rostral part of the CCF (f)
- caudal part of the CCF (g)
- rostral part of the CCF (h)

See Table 1 for a summary of ratios calculated from masks. Owing to size and conformational differences between dogs, raw volume was considered to be an inappropriate measurement and cerebellar volume was therefore evaluated by using nearby structures, against which it was standardised. We expressed relative cerebellar volume as two percentages: as a percentage of the volume of the CCF (Cerebellar Brain Percentage) and as a percentage of the volume of the entire brain (Cerebellar CCF Percentage). These parameters are accepted standards in canine brain volume measurement as they have been used in a study of cerebellar atrophy [22] and CCF parenchyma volume [18], [19]. We used the same percentages to evaluate the volume of the brainstem (Brainstem Brain Percentage and Brainstem CCF Percentage). Cerebellar crowding in the rostral and caudal parts of the CCF was expressed as the percentage of that part of the CCF which was occupied by cerebellum (Caudal Cerebellar CCF Percentage and Rostral Cerebellar CCF Percentage).

**Table 1:** Explanation of the calculation of values.

Cerebellar Brain Percentage	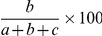
Cerebellar CCF Percentage	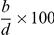
Brainstem Brain Percentage	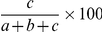
Brainstem CCF Percentage	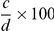
Caudal Cerebellar CCF Percentage	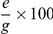
Rostral Cerebellar CCF Percentage	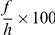

Abbreviations: CCF = caudal cranial fossa. Symbols in equations: a = parenchyma within the cranial cranial fossa, b = cerebellum, c = brainstem, d = CCF, e = cerebellum within the caudal part of the CCF, f = cerebellum within the rostral part of the CCF, g = caudal part of the CCF, h = rostral part of the CCF.

### Statistical analysis

A commercial statistical software package (Prism® for Windows Version 5.00, Graphpad Software Inc, 2007) was used for data analysis. Data was analysed with a one-way ANOVA followed by the Bonferroni multiple comparison test (comparison of the different breed groups) or with an unpaired t-test (comparison of CM and CM/SM groups). Correlations were tested with the Pearson r correlation test. Linear regression was used to model the relationship between variables and ANCOVA was used to determine if the slopes of fitted linear regression models differed significantly. Data are presented as mean ± SEM and p<0.05 was considered significant.

## Results

### Animals

Forty-four small breed dogs (SB), 75 CKCS and 31 Labradors (LD) were compared. The CM/SM and CM groups consisted of 18 and 13 individuals respectively. A list of dog breeds in the SB group can be found in Table 2.

**Table 2:** Number of dogs of each breed included in the Small Breed Dog (SB) group.

Breed	Number of Dogs
Bichon Frise	3
Boston Terrier	2
Bulldog	7
Chihuahua	4
French Bulldog	2
Lhasa Apso	1
Maltese Terrier	2
Minature Poodle	2
Pug	4
Shi Tzu	4
Tibetan Spaniel	1
Toy Poodle	1
Yorkshire Terrier	11

### Morphology

**Figure 3:**
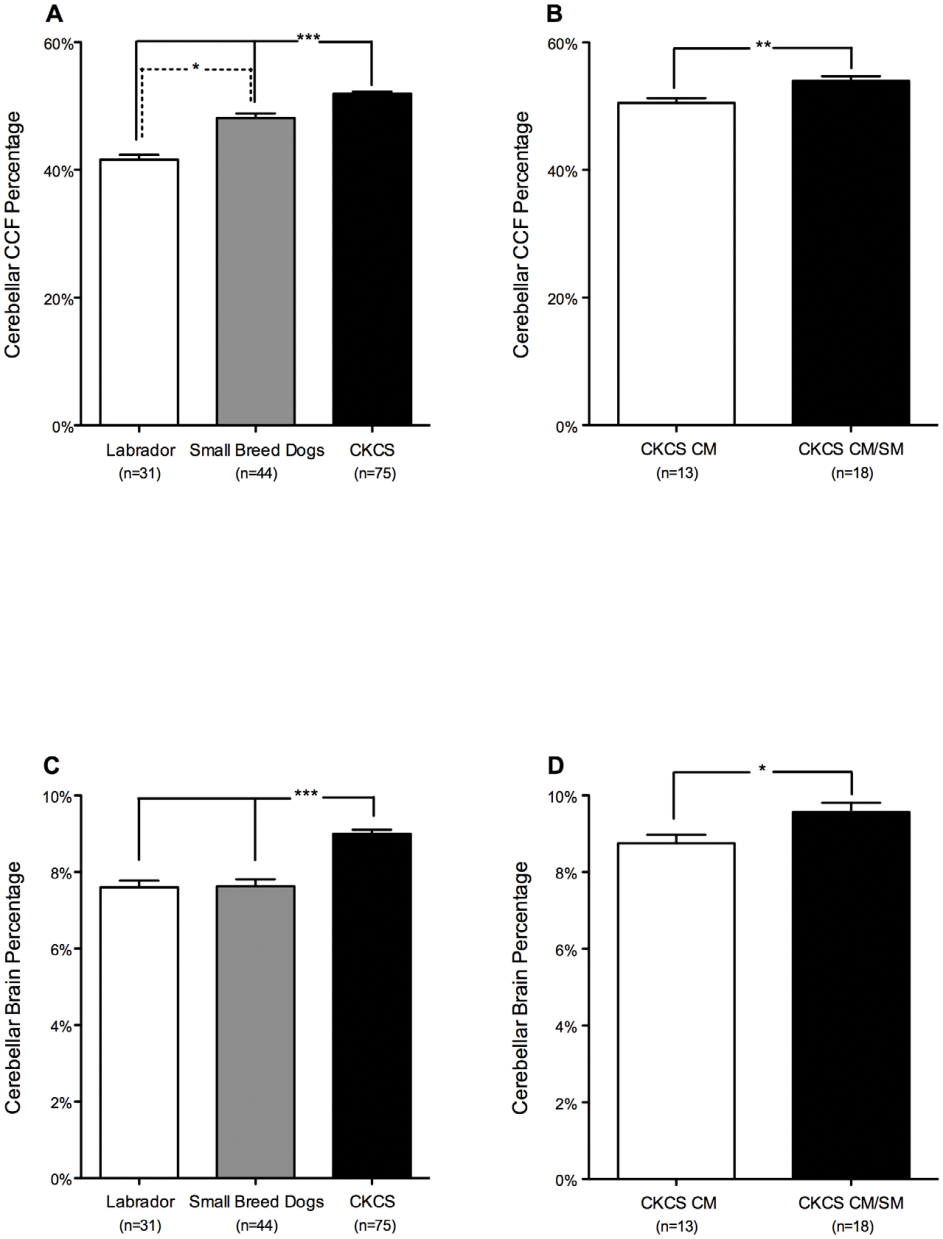
Cerebellar volume. The volume of the cerebellum is expressed as a percentage of the caudal cranial fossa volume (Panels A and B: Cerebellar CCF Percentage) and a percentage of the total brain volume (Panels C and D: Cerebellar Brain Percentage). Cavalier King Charles Spaniels (CKCS) have a relatively larger cerebellum than small breed dogs and Labradors (Panels A and C). CKCS under 2 years of age with Chiari-like malformation (CM) and syringomyelia (SM) (CKCS CM/SM) have a relatively larger cerebellum than CKCS over 5 years of age with CM but without SM (CKCS CM, Panels B and D). *p<0.05, **p<0.01, ***p<0.001.

CKCS had a larger Cerebellar CCF Percentage (CKCS 51.9±0.3% vs. SB 48.1±0.7% [p<0.0001] and LD 41.6±0.8% [p<0.0001]) and Cerebellar Brain Percentage (CKCS 9.00±0.1% vs. SB 7.63±0.2% [p<0.0001] and LD 7.60±0.2% [p<0.0001]) compared to the other groups (Figure 3). The CM/SM group had a significantly larger Cerebellar CCF Percentage (54.0±0.7% vs. 50.5±0.75% [p = 0.0034]) and a significantly larger Cerebellar Brain Percentage (9.56±0.2% vs. 8.75±0.2% [p = 0.0232]) than the CM group (Figure 3). No significant differences were detected between CKCS and other breed groups in Brainstem CCF Percentage (CKCS 37.8±0.3% vs. SB 37.3±0.8% [p = 1.000] and LD 35.8±0.6% [p = 0.062]), however Brainstem Brain Percentage was significantly larger in CKCS than small breed dogs (CKCS 6.55±0.1% vs. SB 5.94±0.2% [p = 0.003] and LD 6.56±0.2% [p = 1.000]). No significant differences were detected between the CM/SM and CM groups in Brainstem CCF Percentage (CM/SM 36.5±0.8% vs. CM 38.5±0.7% [p = 0.0917]) or Brainstem Brain Percentage (CM/SM 6.47±0.2% vs. CM 6.68±0.2% [p = 0.4239]).

**Figure 4:**
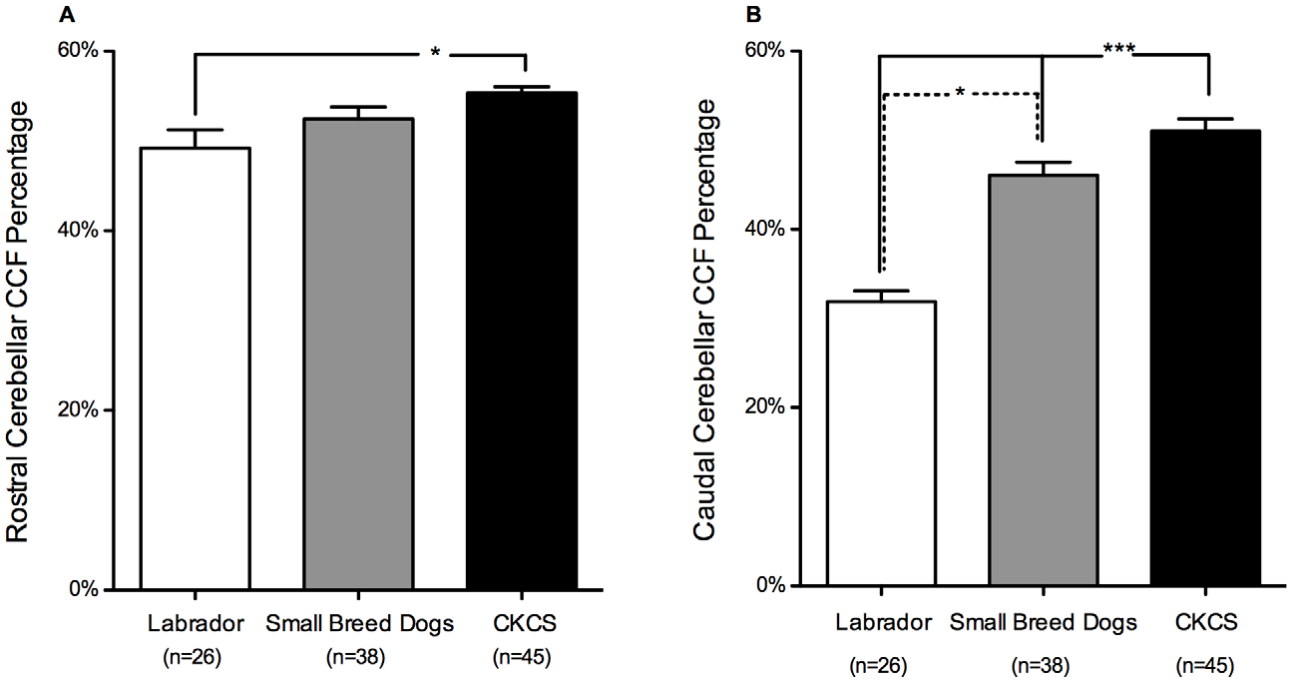
Cerebellar crowding within different parts of the caudal cranial fossa comparing Labradors, small breed dogs and Cavalier King Charles Spaniels. Crowding of cerebellum is defined as the percentage of the volume of each part of the caudal cranial fossa (CCF) which is occupied by cerebellar parenchyma (panel A: Rostral Cerebellar CCF Percentage, panel B: Caudal Cerebellar CCF Percentage). Cavalier King Charles Spaniels (CKCS) have a more crowded rostral CCF than Labradors and more crowded caudal CCF than small breed dogs or Labradors. *p<0.05, ***p<0.001.

**Figure 5:**
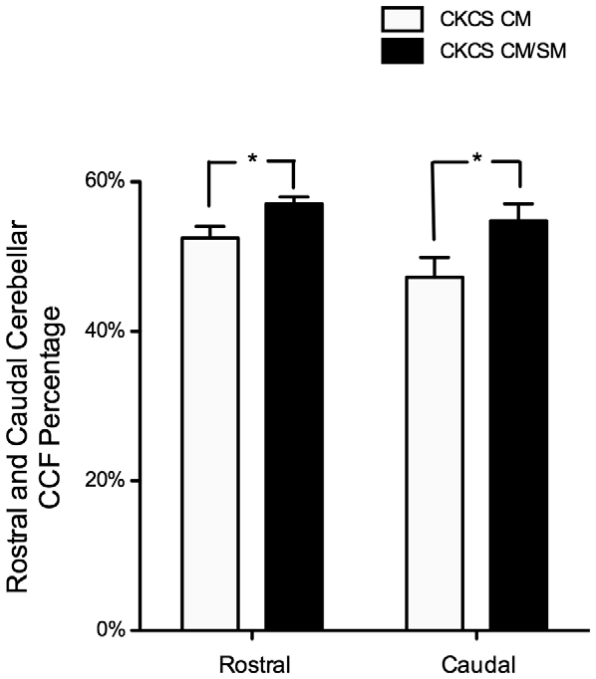
Cerebellar crowding within different parts of the caudal cranial fossa comparing different groups of Cavalier King Charles Spaniels. Crowding of cerebellum is defined as the percentage of the volume of each part of the caudal cranial fossa (CCF) which is occupied by cerebellar parenchyma (Rostral Cerebellar CCF Percentage and Caudal Cerebellar CCF Percentage). CKCS under 2 years of age with Chiari-like malformation (CM) and syringomyelia (SM) (CKCS CM/SM) have a more crowded Rostral CCF and Caudal CCF than CKCS over 5 years of age with CM but without SM (CKCS CM). *p<0.05.

Caudal Cerebellar CCF Percentage was significantly larger in CKCS than the other groups (50.9±1.3% vs. SB 46.1±1.5% [p = 0.033] and LD 31.9±1.2% [p<0.0001]; Figure 4), and also significantly larger in the CM/SM group than the CM group (54.8±2.3% vs. 47.3±2.6% [p = 0.0386]; Figure 5). Rostral Cerebellar CCF Percentage was significantly larger in CKCS (55.3±0.7%) than Labradors (49.2±2.0% [p = 0.004]) but was not significantly larger than small breed dogs (52.5±1.3% [p = 0.282]; Figure 4), and significantly larger in the CM/SM group than the CM group (57.1±0.9% vs. 52.5±1.6% [p = 0.0146]; Figure 5).

**Figure 6:**
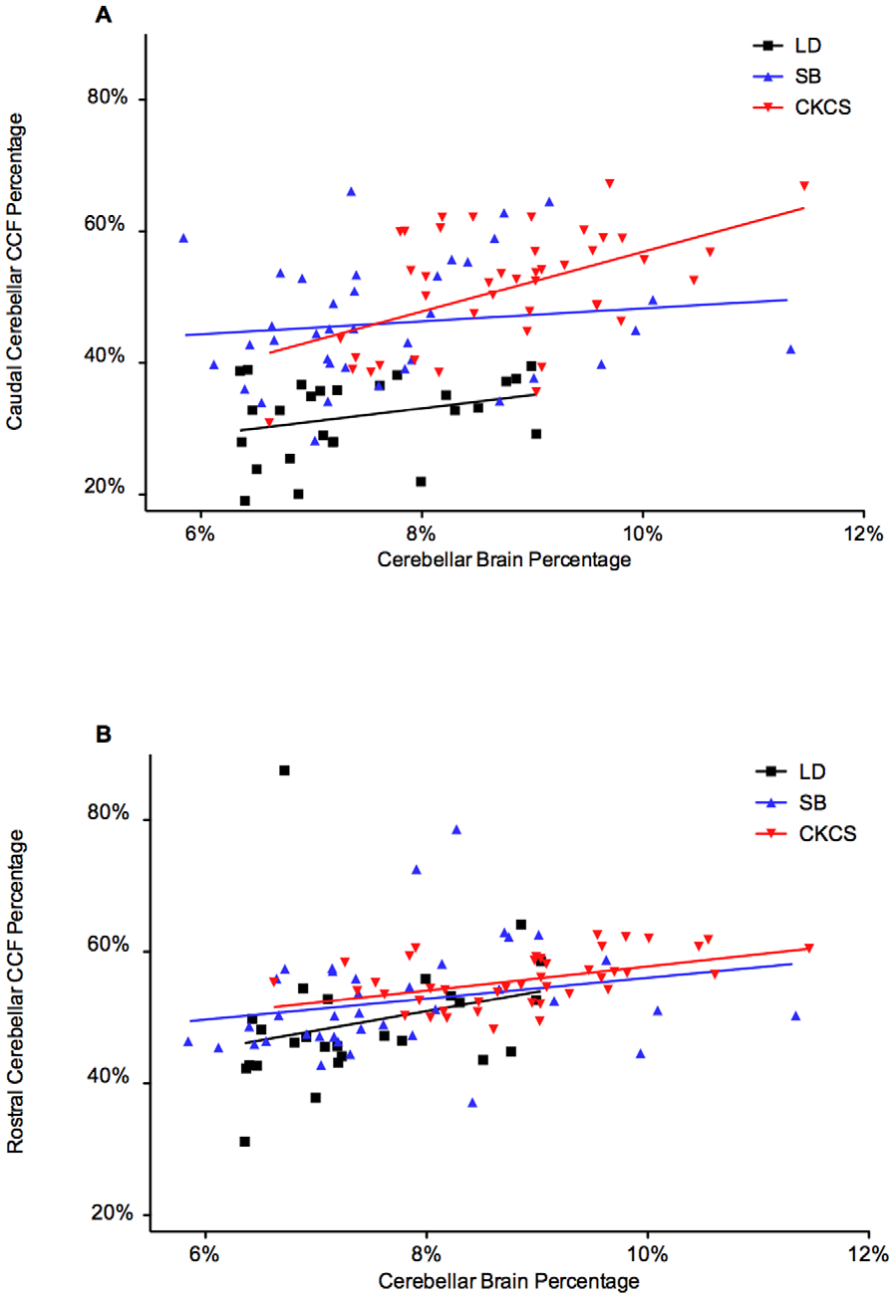
Relationship between Cerebellar Crowding and Cerebellar Volume. Crowding of cerebellum is defined as the percentage of the volume of each part of the caudal cranial fossa (CCF) which is occupied by cerebellar parenchyma (panel A: Rostral Cerebellar CCF Percentage, panel B: Caudal Cerebellar CCF Percentage). The volume of the cerebellum is expressed as a percentage of the total brain volume (Cerebellar Brain Percentage). Fitted linear regression lines are also displayed. Cavalier King Charles Spaniels (CKCS) showed a relationship between cerebellar crowding and volume in both the rostral CCF (p = 0.0008) and caudal CCF (p = 0.0003) and Labradors (LD) and small breed dogs (SB) did not. In CKCS the slope of the fitted model in the caudal CCF was significantly steeper than in the rostral CCF (p = 0.03668), indicating that crowding in the caudal CCF is more sensitive to changes in cerebellar volume.

The relationship between cerebellar overcrowding and relative cerebellar volume was tested with a Pearson r correlation of Caudal Cerebellar CCF Percentage and Cerebellar Brain Percentage: The CKCS group demonstrated a positive correlation (r = 0.5204 [p = 0.0003]), whilst the SB (r = 0.1277 [p = 0.4449]) and LD (r = 0.3016 [p = 0.1342]) group did not. A correlation of Rostral Cerebellar CCF Percentage and Cerebellar Brain Percentage revealed a positive relationship in the CKCS group (r = 0.4886 [p = 0.0008]), but not in the small breed dog (r = 0.2363 [p = 0.1532]) or Labrador (r = 0.2562 [p = 0.2065]) groups. Linear regression was carried out to compare the trend of the rostral CCF and caudal CCF of the CKCS group. An ANCOVA revealed that the fitted linear regression lines have a significantly different slope (rostral CCF 1.872±0.5159, caudal CCF 4.532±1.148 [p = 0.03668], see Figure 6), indicating that in CKCS, crowding of cerebellum in the caudal part of the CCF is more sensitive to changes in the relative volume of the cerebellum than in the rostral part of the CCF. Pearson r correlation between Age vs. Cerebellar CCF Percentage (r = −0.2889, p = 0.1084) and Age. vs. Cerebellar Brain Percentage (r = 0.2801, p = 0.1158) in SM-negative CKCS revealed no statistically significant relationships between relative cerebellar volume and age.

## Discussion

Our results show that the cerebellum is proportionately larger in CKCS when compared to Labradors and small breed dogs and also larger in young CKCS with CM and SM than in older CKCS with CM alone. Furthermore, the degree of cerebellar crowding in the caudal CCF is correlated with increased volume of the cerebellum in CKCS and this is not seen in small breed dogs or Labradors. These findings have implications for the understanding of the pathological mechanisms of CM/SM, and support the hypothesis that it is a multifactorial disease process governed by increased cerebellar volume and failure of the CCF to reach a commensurate size.

### SM develops due to abnormal CSF flow dynamics at the cervicomedullary junction

The presumed pathological mechanism underlying the association between CM and SM is that the development of SM is mediated by abnormal CSF flow dynamics at the cervicomedullary junction [9], [10]. This has been attributed to high-velocity jets of CSF caused by obstruction of the foramen magnum by the herniated portion of the cerebellum and overcrowded brain parenchyma in the caudal occipital region [5] which is hypothesised to set up a hydrostatic pressure differential between the spinal cord and subarachnoid space and results in the accumulation of perivascular fluid which eventually forms a syrinx [9]. The degree of crowding may determine the degree of foramen magnum obstruction, and in turn the tendency for syrinxes to form. Cerebellar volume is potentially a key factor in determining the degree of obstruction and interference in normal CSF flow through the foramen magnum, which disposes dogs to the subsequent development of SM.

### Cerebellar volume is larger in CKCS than in the other breed groups

CM in CKCS is the manifestation of cerebellar herniation through the foramen magnum, and previous studies have shown that this is associated with increased crowding of brain parenchyma within the CCF [18], [19]. In our study we tested the hypothesis that this crowding was due to increased cerebellar volume. Our results show that in CKCS the Cerebellum is more crowded in the caudal CCF than in small breed dogs and Labradors, supporting the theory that CM is due to descent and herniation of the cerebellum through the foramen magnum. CKCS had a relatively larger Cerebellar volume (and a similar brainstem volume) when compared to small breed dogs and Labradors, supporting hypothesis i) (that CKCS have increased cerebellar volume compared to other breeds of dog).

### Uniquely in CKCS, increased cerebellar volume is correlated with crowding in the caudal CCF

Our results support hypothesis iii) which states that in CKCS an increase in relative cerebellar volume is correlated with an increase in cerebellar crowding in the caudal CCF. It should be noted that small breed dogs and Labradors do not show the same relationship. We infer from this result that during cranial development in Labradors and small breed dogs, a compensatory mechanism maintains the relationship between cerebellar volume and CCF dimensions, and this mechanism is defective in CKCS. We also found in CKCS that cerebellar crowding in the caudal CCF is more sensitive to changes in relative cerebellar volume than cerebellar crowding in the rostral CCF, which is consistent with the theory that increased cerebellar volume results in the cerebellum shifting caudally and causes obliteration of dead space in the caudal CCF. This also causes herniation of the cerebellum through the foramen magnum (i.e. CM). This finding is consistent with previous research, which has shown that CKCS possess a CCF parenchyma proportionately similar in volume to that of a Labrador and a CCF volume similar to small breed dogs, suggesting that CCF growth in CKCS is not keeping pace with the growth of brain parenchyma [18]. This is an interesting finding as it suggests that CM/SM may be a multifactorial disease dependent on the cumulative effects of a small CCF and large cerebellum.

### Increased cerebellar volume is associated with Syringomyelia in CKCS

In this study we found that CKCS under the age of 2 with SM (the CM/SM group) have an increased cerebellar volume when compared to CKCS over the age of 5 without SM. Unlike humans, we found that CKCS do not appear to have age-related atrophy as there was no correlation between relative cerebellar volume and age. This supports hypothesis ii), that increased cerebellar volume in CKCS is associated with syringomyelia. Previous volumetric studies in CKCS have shown that there is an association between SM and CCF parenchyma volume [19], [26], but this is the first time that Cerebellar volume has been linked to SM. The cerebellum to brain volume ratio is consistent between normal dogs and has been shown to decrease with cerebellar degenerative disorders [22], but it has never been shown to be increased in size in a canine neurological disorder. To the authors' knowledge, no studies have examined the role of cerebellar volume in human Chiari malformation I and associated SM.

### Conditions leading to increased brain volume in humans

Generalised overgrowth of brain parenchyma (megalencephaly) is recognised in over 100 human syndromes [27] and in some of these conditions posterior cranial fossa overcrowding and herniation of the cerebellum have been reported. These include Macrocephaly–Capillary Malformation, also known as macrocephaly-cutis marmorata telangiectatica congenital [28], [29], the closely related megalencephaly polymicrogyria-polydactyly hydrocephalus (MPPH) syndrome [30], Rasopathies [31], Alexander's disease [32] and Lhermitte-Duclos disease [33]. Megalencephaly is suggestive of disorders of generalized neuro- and gliogenesis. Some of these conditions are also manifested in different parts of the body, such as Macrocephaly–Capillary Malformation, a poorly understood syndrome which is characterized by malformed capillaries and prenatal somatic overgrowth with numerous asymmetries [28], [29]. Other common causes of megalencephaly include Rasopathies, a group of diseases caused by genetic mutations which lead to an upregulation of the mitogen-activated protein kinase (Ras/MAPK) pathway [31]. Affected individuals have multiple defects including progressive brain overgrowth which is caused by proliferation of cortical progenitor cells and premature gliogenesis. A number of Rasopathies which can result in cerebellar herniation include Costello's syndrome [34], Neurofibromatosis type 1 [35], Noonan's syndrome [36] and cardio-facio-cutaneous syndrome (CFC) [37].

### Conditions leading to increased cerebellar volume in humans

Generalised megalencephaly in the CKCS is not supported by our findings which indicate that there is enlargement of the cerebellum relative to overall brain volume. However, there are only a few rare syndromes of cerebellar overgrowth in humans which do not involve generalised brain overgrowth. Diffuse cerebellar enlargement (macrocerebellum) is a poorly defined syndrome proposed to be related to the response of the cerebellum to an over-expression of growth factors to augment slow cerebral growth [38]. Primary defects of the cerebellum include Lhermitte-Duclos disease [33], in which a slow-growing harmatoma causes diffuse hypertrophy of the cerebellar stratum granulosum. Chiari Malformation II in humans, although associated with reduced posterior cranial fossa volume, is thought to be neuroectodermal in origin and involves enlargement of the anterior lobes of the cerebellum [13], [39]. The possibility that the pathogenesis of CM in CKCS is due to a neuroectodermal defect should be investigated. The authors are currently investigating the possibility of neurological deficits in CKCS which are referable to cerebellar dysfunction (including a specific “puppy-like” ataxia). Scope for further research would involve histological and embryological studies of the forebrain and cerebellum to evaluate possible developmental disorders.

### Bony development of the CCF

It has been proposed that the volume mismatch between the CCF and brain parenchyma in CKCS can be explained by impaired occipital bone development and the consequent reduction in CCF volume [5], [16], [21]. A recent volumetric study of CKCS has found that individuals in the CM/SM group have a minimally smaller CCF volume than individuals in the CM group [26]. In this study, we find that in CKCS, unlike small breed dogs or Labradors, there is a positive correlation between the volume of the cerebellum and degree of crowding in the caudal CCF, which suggests that CM may be due to CCF development not keeping pace with growth of the cerebellum. This supports the idea that CM/SM in CKCS may in fact be multifactorial and an abnormal development process affecting the CCF may be acting as a disease modifier. Although the occipital bone comprises a single bony plate in an adult individual, its development is complex and mosaic as it develops from the basioccipital, exoccipital, and supraoccipital bones which are derived from distinct acrochordal and parachordal, occipital arch, and supraoccipital cartilages, respectively [40]. Impaired CCF development may be caused by a failure of communication between one or more of these progenitors and the developing neural tube (specifically, rhombomere 1, which gives rise to the cerebellum) [41], [42]. Alternatively, it could simply be explained by premature closure of growth plates between the bones of the CCF, as has been reported in humans [14]. It has been found that pre-natal posterior cranial fossa development in humans is independent of cerebellar volume but closely parallels the development of the supratentorial bony compartment [43]. If this is also true in dogs it may have implications for the development of CM, as the CCF may have a restricted capacity to adapt to the volume of an enlarged cerebellum through expansion of the sutures between the occipital bones and their neighbours.

### Occipital bone resorbtion

Studies of human skulls have found that the occipital bones adapt to the shape of the growing cerebellum. In one study, the occipital bones showed a resorbtive pattern of bone around the cerebellar hemispheres in adults and in children [44], suggesting that bone remodelling continues long after skull sutures have fused. It has also been noted on post-mortem examination of CKCS and other small breed dogs that the supraoccipital bone overlying the cerebellar vermis is remarkably thin and sometimes eroded so that the foramen magnum is enlarged dorsally [23], which could indicate that there has been substantial bone resorbtion. Work is needed to elucidate the mechanisms of occipital growth in dogs to determine the extent to which an osteo-resorbtive process can mitigate an enlarged cerebellum in CKCS and in other breeds.

### Scope for further research

Histopathological studies of occipital bone development are needed in order to compare the CKCS to other breeds of dog. The possibility of a cerebellar growth disorder also deserves scrutiny. In order to assess the clinical significance of cerebellar volume and CCF volume as prognostic indicators, further cohort and longitudinal studies are needed. In human medicine, investigation of the role of increased cerebellar volume in the development of Chiari malformation I and associated SM may be warranted.

### Conclusions

Our findings show that the CKCS has a relatively larger cerebellum than small breed dogs and Labradors and there is an association between increased cerebellar volume and SM in CKCS. In contrast to small breed dogs and Labradors, CKCS exhibit correlation between increased cerebellar volume and cerebellar crowding within the caudal CCF, suggesting that CCF growth in CKCS is not keeping pace with the growth of the cerebellum.
